# Functionalized graphene as a model system for the two-dimensional metal-insulator transition

**DOI:** 10.1038/srep19939

**Published:** 2016-02-10

**Authors:** M. S. Osofsky, S. C. Hernández, A. Nath, V. D. Wheeler, S. G. Walton, C. M. Krowne, D. K. Gaskill

**Affiliations:** 1 Naval Research Laboratory, Washington, DC, USA; 2 George Mason University, Fairfax, VA, USA

## Abstract

Reports of metallic behavior in two-dimensional (2D) systems such as high mobility metal-oxide field effect transistors, insulating oxide interfaces, graphene, and MoS_2_ have challenged the well-known prediction of Abrahams, *et al.* that all 2D systems must be insulating. The existence of a metallic state for such a wide range of 2D systems thus reveals a wide gap in our understanding of 2D transport that has become more important as research in 2D systems expands. A key to understanding the 2D metallic state is the metal-insulator transition (MIT). In this report, we explore the nature of a disorder induced MIT in functionalized graphene, a model 2D system. Magneto-transport measurements show that weak-localization overwhelmingly drives the transition, in contradiction to theoretical assumptions that enhanced electron-electron interactions dominate. These results provide the first detailed picture of the nature of the transition from the metallic to insulating states of a 2D system.

The excitement generated by the achievement of metallic single layer graphene has obscured the fact that seminal theoretical work predicted that purely two-dimensional (2D) systems should not be metallic^1^. A possible explanation for the metallic behavior in graphene is that massless Dirac electrons exhibit Klein tunneling and are thus, immune to the effects of disorder^2^,^3^. This argument is contradicted by reports that the carriers often have mass^4^,^5^,^6^, possibly due to disorder and/or the underlying substrate breaking lattice symmetry or the fact that the Fermi energy is far from the Dirac point^2^. Thus, graphene should be described by the theory presented in reference 1 if there is disorder in the potential binding the electrons. The situation is confounded further by later theoretical work showing that Dirac Fermionic systems with no spin-orbit interactions and Gaussian correlated disorder exhibit scaling behavior but should always be metallic^3^. The observed metallic behavior is an unquestionable addition to a series of systems such as high mobility metal-oxide field effect transistors (HMFET)^7^ and interface oxides^8^ that have demonstrated a 2D metallic state (although the nature of that state for the HMFET’s is not well understood). These systems are presumed to be 2D due to their geometry but might have some three-dimensional character since the charge regions extend over finite distances^9^,^10^ that could explain their metallic transport properties. The experimental conditions are also confounded by the fact that the thickness and shape of the charge layer varies with the application of a gate voltage. In contrast, graphene is a model system for studying the 2D metal-insulator transition (MIT) as it is a pure 2D system (with a constant thickness of 0.335 nm) like MoS_2_ (which has recently been shown to also have an MIT^11^,^12^,^13^,^14^). In this work, we increase the resistivity of epitaxial graphene through surface functionalization by exposure to low energy plasmas. These results reveal the existence of a 2D MIT in epitaxial graphene where the pre-functionalization values of carrier concentrations and mobilities are ~10^12^–10^13^ cm^−2^ and ~700–900 cm^2^V^−1^s^−1^, values that are out of the range of applicability for the models developed to describe the previous results on the HMFETs^15^,^16^ where the disorder is thought to be screened by high mobility electrons. Recent theoretical work treated the transition density for the apparent MIT observed in the HMFETs^17^. Since these models treat a transition that occurs at finite temperature, rather than the true MIT quantum phase transition that occurs at T = 0, they are not applicable to this work. It is possible that a more recent general scaling model that was developed for the high mobility case, and allows for the existence of a 2D MIT^18^, can be used to model the graphene system as well. The results presented here demonstrate that the strongly localized state is separated from the metallic state by a weakly localized phase with conductivity, σ, ~log(T) similar to results recently reported for thin films of RuO_2_^19^.

Previous work has shown that an MIT does indeed exist for graphene: it is well established that graphene can have a metallic state and Chen, *et al.* have demonstrated that insulating samples result through exposure to ion damage^20^. Furthermore, Bostwick, *et al.* observed an MIT by showing a large increase in room temperature resistance accompanied by a breakdown of the quasi-particle description as determined from photoemission and electronic transport measurements in graphene exposed to atomic hydrogen^21^. This report and one by Withers, *et al.* on fluorinated graphene transistors^22^ demonstrated R(T) behavior that was consistent with 2-d variable range hopping (VRH).

Theoretical work described how the Anderson localized state can form in hydrogenated graphene^23^. Key to understanding the 2D MIT is the study of metallic transport near the transition. In 3D materials, it is known that weak-localization (WL) and enhanced electron-electron interactions (EEI) control the metallic transport properties near the MIT. For metallic graphene with moderately high mobility, there have been several studies reporting WL and/or EEI^24^,^25^,^26^,^27^,^28^,^29^,^30^,^31^,^32^. Those results, while suggestive, are for graphene relatively far from the MIT where WL and EEI can be treated as corrections to the conductivity. That approach fails near the MIT, a quantum phase transition, where scaling models of phase transitions are needed to describe the properties^33^,^34^. Thus, it appears that a scenario analogous to the three-dimensional case where the disorder driven MIT is described by a phase diagram with four regions^35^,^36^: insulating, critical, amorphous metal, and conventional metal can be observed for the 2D case. In the present study the systematic increase in the graphene’s sheet resistance resulting from exposure to low energy plasmas has been used to determine a critical exponent of this phase transition and estimate the relative contributions of WL and EEI as the strongly localized phase is approached.

## Preparing and functionalizing epitaxial graphene

Several samples of epitaxial graphene were grown via Si sublimation from nominally on-axis SiC (0001) substrates^37^. Prior to graphene growth, substrates were etched in hydrogen at 1520 °C, 100 mbar for 10–30 min. to remove polishing damage. Graphene was then synthesized in 10 standard liters per minute of Ar at 1540 °C, 100 mbar for 25–35 min. These conditions resulted in graphene with an average thickness of 1.5 layers over a 400 μm diameter area as determined by x-ray photoelectron spectroscopy. While this analysis indicates that part of the samples are double layer graphene which has been demonstrated to have an MIT^38^,^39^, previous work on graphene grown under these conditions demonstrated that the graphene was mostly single layer on a terrace with 2 or 3 layers on the step edges^37^, consistent with the delamination of the buffer layer on the step facets^40^. The samples were then fashioned into a pattern that enabled standard four-probe resistivity and Hall measurements (supplementary information). Each sample was then systematically exposed to electron beam generated plasmas produced in mixtures of O_2_, SF_6_, or N_2_ to introduce oxygen-, fluorine-, or nitrogen-functional groups^41^,^42^. Some samples were also selectively exposed to a vacuum anneal after plasma treatments to reduce the resistance. Increasing dosage is indicated by an increasing numerical symbol, i.e., N0 (Nitrogen series, no dose), N1, N2, etc.; see Table S1 for details. Raman measurements indicated that the graphene signature was present after functionalization (supplementary information).

**Figure 1:**
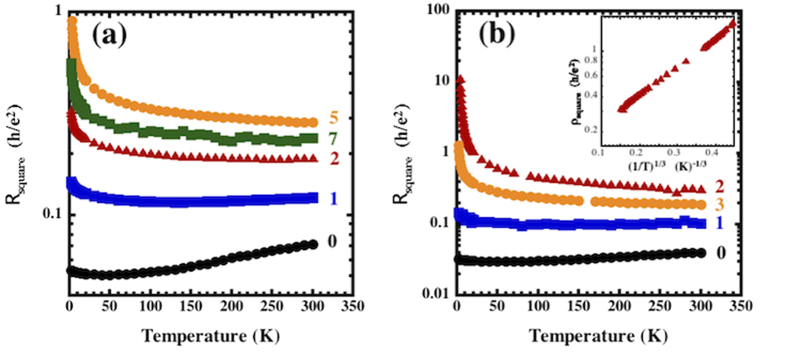
Resistance/square (plotted in units of h/e^2^) for graphene exposed to (a) nitrogen- and **(b)** oxygen-containing plasmas. Estimates for the total ionized species produced for each sample are shown in Table S1. Inset (**b**): Log(resistance/square) vs. (1/T)^1/3^, the behavior expected for 2D variable range hopping, for oxygen sample 2. The curves denoted by “0” are for untreated graphene while the curves denoted “1–7” correspond to increasing plasma dose, with values given in Table S1 (supplementary information).

As grown, the samples had resistance, R, ~1000 Ω/□, carrier concentrations of ~10^12^–10^13^ cm^−2^, and mobilities ~700–900 cm^2^V^−1^s^−1^ measured at room temperature (supplementary information). It is important to note that very low currents were used for the transport measurements to ensure that local heating did not obscure the results at low temperature (supplementary information). The carrier concentrations are comparable to those reported for HMFET’s (~10^10^–10^12^ cm^−2^)^43^ with the starting mobility values higher than those reported for oxide interface FET systems^44^,^45^,^46^, and smaller than those reported for conventional HMFET devices, ~10^4^ cm^2^V^−1^s^−1 ^43. By exposing the graphene to the plasmas, the room temperature resistance eventually increased to values that exceed the quantum resistance, h/(2e^2^). The samples can thus be driven through the MIT by systematically exposing the graphene to increasing plasma doses and vacuum anneals (Fig. 1). The amount of induced disorder varied by element with F and O having the strongest influence (supplementary information).

## Transport properties: 2D metal-insulator transition

Figure 1a,b show data for N and O exposures clearly demonstrating transitions from conventional metallic behavior, dR/dT > 0, for dose 0 and 1 (with low temperature deviations) to a state with dR/dT < 0 for higher doses. At the highest oxygen doses the graphene exhibits behavior consistent with 2D variable range hopping (VRH), R ~ exp(1/T^1/3^), demonstrating the transition to a strongly localized insulating state (inset Fig. 1b). The data for lower O exposures and for the N exposures did not show VRH behavior. Fluorine was so effective in increasing the resistance that only a few R(T) curves could be obtained before the strongly localized state was achieved. We note that our unfunctionalized graphene exhibits evidence of localization corrections at low temperature that are probably due to defects introduced during growth. Since pristine, extremely high mobility graphene is known to be metallic, it is evident that an MIT exists in this system.

**Figure 2:**
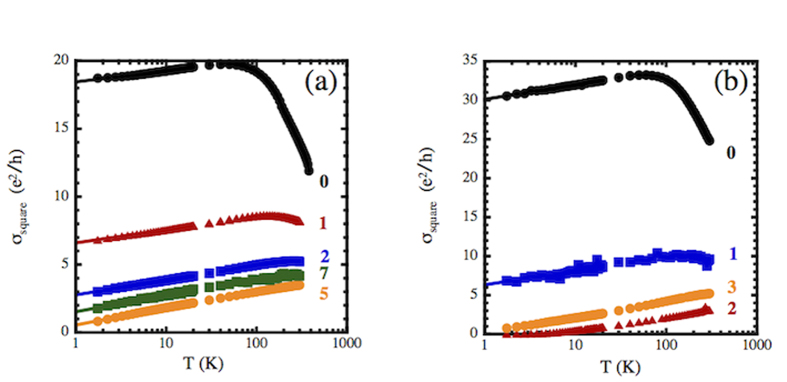
Conductivity per square plotted vs. log(T) as a function of dose for the data in Fig. 1 for plasma exposures to (**a**) nitrogen and (**b**) oxygen. The solid lines are extrapolated fits to σ = σ_0_ + σ_1_log(T) for T < 10 K.

The original theoretical work on WL that described the three dimensional MIT^1^ also predicted that all 2D systems will be insulators with σ ~ log(T). Later work indicated that this log(T) behavior would also result from enhanced electron-electron interactions in diffusive 2D systems^47^. Indeed, work on Si MOSFETS^48^,^49^ and very thin films demonstrated this log(T) behavior^50^,^51^,^52^,^53^,^54^. Figure 2 shows plots of conductance as a function of log(T) for the data in Fig. 1. These curves clearly show these samples having 2D transport characteristics at low temperatures. The relevant low temperature data were fit to those fits are shown as solid lines in Fig. 2.


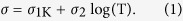


One of the key issues in understanding the MIT is the slope of the critical phase line, or “mobility edge,” that describes the transition into the strongly localized state. In three dimensions, this line is usually defined as the relationship between a driving parameter, generically labeled as p, and 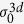, the value of conductivity extrapolated to T = 0^33^,^34^,^35^,^50^,^55^. The usual formulation is 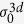 ~ (p − p_c_)^ζ^ where p_c_ is the critical value of p (where 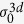 = 0) and where ζ is a critical exponent^33^,^34^,^35^,^56^. Experimentally, p is often the carrier concentration. Another choice for p is the bare, high-energy conductivity that can be approximated by the room temperature conductivity^55^. It has been shown that in three dimensions ζ = 1/2 in Si:P^57^,^58^ while ζ = 1 in disordered metals^33^,^34^,^50^,^55^

**Figure 3:**
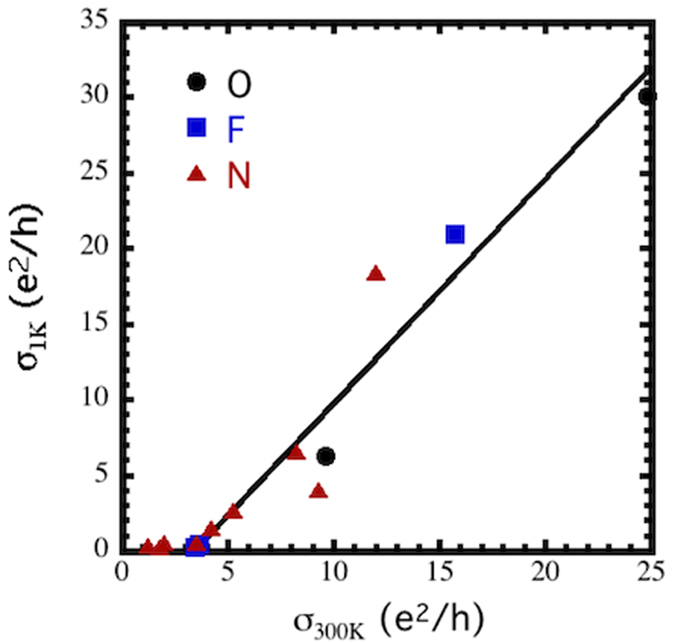
Conductivity at 1 K vs. conductivity at 300 K showing the continuous nature of the conductivity as the sample approaches the MIT similar to the linear “mobility edge” observed in many three dimensional systems. The line is a linear fit to the data for σ_1K_ > 0. F, O, and N refer to samples subject to plasmas containing SF_6_, O_2_, and N_2_, respectively.

In two dimensions, the data analysis is complicated by the fact that the data cannot be extrapolated to T = 0 since σ has a log(T) behavior. In this case, one can obtain an analog of the mobility edge by replacing 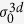 with σ_1K_ from equation 1 so that the relevant expression becomes σ_1K_ ~ (σ_300K_ − σ_c_)^ζ^ where σ_c_ is the value of σ_300K_ for which σ_1K_ = 0. If the phase transition is governed by a scaling law this formulation should capture the nature of the transition (*i.e.* whether it is continuous and, if so, the value of ζ). This mobility edge is plotted in Fig. 3 using data from the three types of exposures. The plot clearly shows that the transition is continuous with ζ = 1, similar to many disordered 3-d systems.

## Weak-localization vs. enhance electron-electron interactions

There have been several theoretical approaches for describing diffusive transport in disordered 2D conductors using scaling^33^,^59^,^60^,^61^,^62^. These models predict insulating behavior in 2D. However, they require the suppression of WL, either by strong spin-flip scattering, strong spin-orbit coupling, or a strong internal magnetic field (e.g., in a ferromagnet) leaving EEI as the relevant phenomenon near the MIT.

Magneto-transport measurements, including the Hall resistance, provide a means to distinguish between the contributions of WL and EEI to conductance. Previous work on samples far from the insulating phase has demonstrated a wide variety of behaviors that include WL and/or EEI corrections to the conductance^24^,^25^,^26^,^27^,^28^,^29^,^30^,^31^,^32^. None of those studies considered samples close to the strongly localized state.

**Figure 4:**
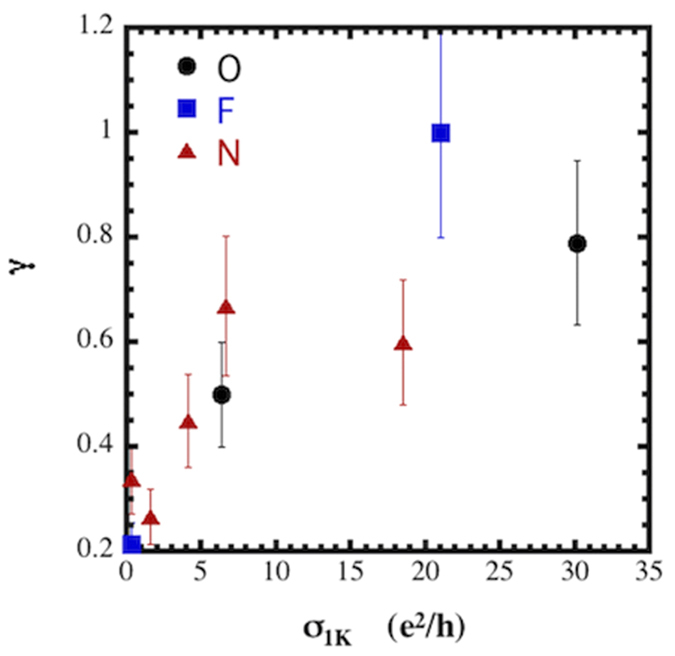
The ratio γ = (ΔR_Hall_/R_Hall_)/(ΔR/R) at 1.75 K as a function of σ_1K_, a measure of the distance to the exponentially localized state.

For our functionalized samples the Hall resistance, R_Hall_ showed a log(T) temperature dependence at low temperatures (supplementary information). This behavior is consistent with that described by Altshuler and Aronov for EEI in disordered systems^47^


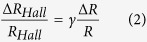


where R_Hall_ is the Hall resistance, R is the resistance, and γ = 0 for no EEI^63^, γ = 2 for EEI, and γ > 2 if there is spin-orbit interaction. The values of γ at 1.75 K were determined from the slopes of the R(T) and R_Hall_(T) data below 10 K. These results are plotted in Fig. 4 as a function of σ_1K_, a measure of the distance to the exponentially localized state. It is apparent that γ < 2 with a clear trend in which γ approaches 0.2 as the system approaches the exponentially localized state. This is in contrast to the results reported by Lara-Avila, *et al.*^24^ who found γ ≥ 2. The source of this discrepancy may be the fact that the mobilities of the samples studied in ref. 24 were ~6000–7000 cm^2^/(V-s) which is 10–100 times larger than those measured in this work and indicate the measurements were far from the MIT. The systematic decrease in γ as our system approaches the strongly localized phase is similar to behavior observed in Si MOSFETS where γ ~ 2 for low channel resistance but approached 1 as the channel resistance increased^64^. Thus, the Hall resistance results show that the influence of EEI decreases as the system approaches the strongly localized phase and that transport properties are dominated by WL.

**Figure 5:**
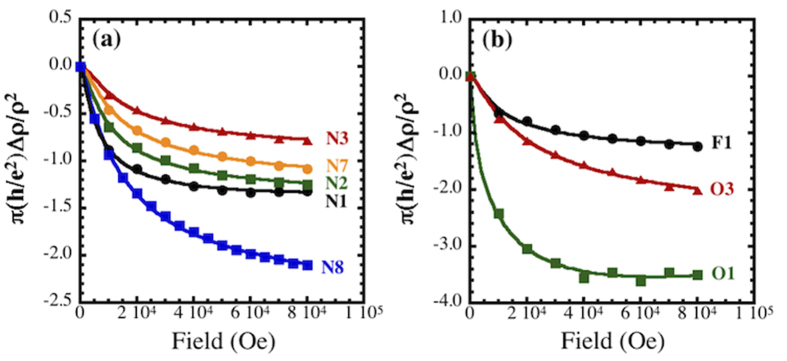
Magneto-resistance data obtained at 1.75 K for samples treated in (**a**) nitrogen- and (**b**) oxygen- and fluorine-containing plasmas. Exposures and σ(1 K) for the doses are listed in Table S1. The solid symbols are experimental data and the solid lines are fits to the weak-localization model of McCann, *et al.* (ref. 65).

Magneto-resistance (MR) measurements, which are also influenced by WL and EEI, were performed simultaneous to the Hall measurements to further explore how they influence transport. Figure 5 shows the MR results at 1.75 K for various plasma exposures plotted in the manner suggested by the theory of McCann, *et al.*^65^. In that theory, the expression for the MR, 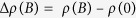, is


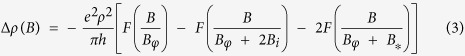


where *F* is a function containing the natural logarithm and the digamma function,


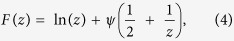


and ρ is the resistivity. Subscripted magnetic fields in equation 3 are simply the effective magnetic representations of the relaxation times,


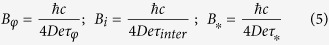


where 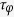 and 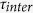 are the relaxation times for inelastic decoherence and intervalley scattering, respectively, and intravalley scattering and trigonal warping are folded into intervalley scattering through


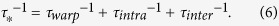


We note that the curves in Fig. 5 are for ρ_xx_ data. Formally, the inverse of the conductivity tensor should be used^25^ but the contribution of ρ_xy_ is negligible and can be ignored. The plots clearly have the shape and negative MR that is characteristic of WL. In contrast, for EEI the MR is characterized by a B^2^ magnetic field dependence^25^,^31^,^66^ and is usually positive^33^. The solid lines in Fig. 5 are fits to equation 3. The characteristic time scales resulting from the fits are shown in the supplementary information. While this model appears to provide a good fit to the data, it must be emphasized that the values of the parameters extracted should not be taken too seriously since the theory describes a correction to resistance of a weakly disordered metal, a situation far from that of graphene close to the transition to the strongly localized state that is described here. The crucial finding is that, consistent with the Hall data, the MR results indicate that WL is the dominant transport phenomenon, contradicting the assumptions of the prevailing theoretical treatments of 2D disordered systems that treat EEI as the dominant mechanism influencing transport^33^,^59^,^60^,^61^,^62^.

More recently, Dobrosavljević, *et al.*^18^ extended the theory of Abrahams, *et al.*^1^ to include electron-electron interactions (for screening but not EEI) by relaxing the assumption that the scaling function is monotonic and negative for “large” conductance. This modification results in the prediction of a 2D MIT. To explore whether this theory can describe our results, we use the scaling model conductance from ref. 18:


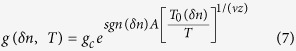


where *g*_*c*_ is the critical conductivity for the MIT, sgn( ) is the sign operator, 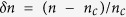 with *n* and *n*_*c*_ the carrier and critical carrier concentration, *T*_*0*_ is a crossover temperature which has 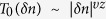, *A* is a dimensionless constant of order unity, ν is the correlation length exponent, and *z* is the dynamical exponent relating temperature and length scale, *L,* by 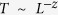. By expanding the exponential to two terms, expanding the natural logarithm, combining terms, and dropping the A factor, we obtain


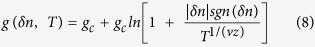


Near the critical point (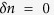), and neglecting the sgn operator for the moment, generates the following formula:


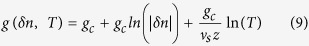


which is consistent with the weak localization approach discussed earlier, and the use of equation (1) to fit our data. The relationship between ν and ν_*s*_ is 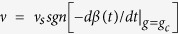, 
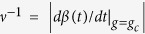
, with ν > 0, where *β* is the scaling function from reference^18^. Since *β* is non-monotonic, its derivative would have to have the sign that results in ν_*s*_ > 0 for our case.

While this model appears to describe the graphene results, it must be noted that the original motivation for this theory was the discovery of an apparent 2D MIT in HMFET’s. It isn’t clear that the theory is applicable here, especially since it also predicts that these 2D systems are perfect metals that are not Fermi liquids in the metallic state, a description that does not apply to graphene. It is possible that there is a rich phase space that encompasses both the low and high mobility cases that should be pursued further although it is not clear that this model can be used to quantitatively analyze the data. Such an analysis will probably require a more detailed two-parameter scaling model that explicitly includes EEI and WL.

In conclusion, we have demonstrated the existence of a continuous transition to a strongly localized state in graphene, a model 2D electronic system that is known to be metallic in its pristine state (i.e. exfoliated flakes). These results contradict a theoretical analysis that predicts robust metallic behavior in graphene and clearly show that the phase diagram is analogous to that for three dimensions with the conductivity having a log(T) temperature dependence rather than T^1/2^ dependence above the strongly localized phase. Magnetoresistance and Hall resistance measurements reveal that WL dominates as the strongly localized state is approached, contrary to the assumptions of renormalization group theories that only treat EEI to describe 2D disordered systems and do not predict a 2D MIT. These results are consistent with a scaling model by Dobrosavljevic ´, *et al.* that predicts an MIT in two dimensions and suggests that a more complete theory is needed for the 2D MIT.

## Additional Information

**How to cite this article**: Osofsky, M. S. *et al.* Functionalized graphene as a model system for the two-dimensional metal-insulator transition. *Sci. Rep.* **6**, 19939; doi: 10.1038/srep19939 (2016).

## Supplementary Material

Supplementary Information

## References

[b1] Abrahams E., Anderson P. W., Licciardello D. C. & Ramakrishnan T. V.. Scaling theory of localization: Absence of quantum diffusion in two dimensions. Phys. Rev. Lett. 42, 673–676 (1979).

[b2] Castro Neto A. H., Guinea F., Peres N. M. R., Novoselov K. S. & Geim A. K.. The electronic properties of graphene. Rev. Mod. Phys. 81, 109–162 (2009).

[b3] Das Sarma S., Adam S., Hwanf E. H. & Rossi E.. Electronic transport in two-dimensional graphene. Rev. Mod. Phys. 83, 407–470 (2011).

[b4] Fuhrer M. S.. Critical Mass in Graphene. Science 340, 1413–1414 (2013). 23788788 10.1126/science.1240317

[b5] Hunt B. *et al.* Massive Dirac Fermions and Hofstadter Butterfly in a van der Waals Heterostructure. Science 340, 1427–1430 (2013). 23686343 10.1126/science.1237240

[b6] Tiras E. *et al.* Effective mass of electron in monolayer graphene: Electron-phonon interaction. J. Appl. Phys. 113, 043708 (2013).

[b7] Kravchenko S. V., Kravchenko G. V., Furneaux J. E., Pudalov V. M. & D’Iorio M.. Possible metal–insulator transition at B = 0 in 2 dimensions. Phys. Rev. B 50, 8039–8042 (1994). 10.1103/physrevb.50.8039 9974805

[b8] Ohtomo A. & Hwang H. Y.. A high-mobility electron gas at the LaAlO_3_/SrTiO_3_ heterointerface. Nature 427, 423–426 (2004). 14749825 10.1038/nature02308

[b9] Mishra U. K. & Singh J.. Semiconductor device physics and design (Springer, The Netherlands, 2008).

[b10] Krowne C. M. & Holm-Kennedy J. W.. Energy relaxation of electrons in the (100) n-channel of a Si-MOSFET: II. Surface phonon treatment, Surface Science 46, 232–250 (1974).

[b11] Baugher B. W. H., Churchill H. O. H., Yang Y. & Jarillo-Herrero P.. Intrinsic Electronic Transport Properties of High-Quality Monolayer and Bilayer MoS_2_. Nano Lett. 13, 4212–4216 (2013). 23930826 10.1021/nl401916s

[b12] Radisavljevic B. & Kis A.. Mobility engineering and a metal–insulator transition in monolayer MoS_2_. Nat. Mat. 12, 815–820 (2013). 10.1038/nmat3687 23793161

[b13] Schmidt H. *et al.* Transport Properties of Monolayer MoS_2_ Grown by Chemical Vapor Deposition. Nano Lett. 14, 1909–1913 (2014). 24640984 10.1021/nl4046922

[b14] Chen X. *et al.* Probing the electron states and metal-insulator transition mechanisms in molybdenum disulphide vertical heterostructures, Nat. Commun. 6:6088, 10.1038/ncomms7088. 25586302

[b15] Punnoose A. & Finkel’stein A. M.. Metal–insulator transition in disordered two-dimensional electron systems. Science 310, 289–291 (2005). 16224015 10.1126/science.1115660

[b16] Anissimova S., Kravchenko S. V., Punnoose A., Finkel’stein A. M. & Klapwijk T. M.. Flow diagram of the metal–insulator transition in two dimensions. Nature Phys. 3, 707–710 (2007).

[b17] Das Sarma S. & Hwang E. H.. Two-dimensional metal-insulator transition as a strong localization induced crossover phenomenon, Phys. Rev. B 89, 235423 (2014).

[b18] Dobrosavljević V., Abrahams E., Miranda E. & Chakravarty S.. S. Scaling theory of two-dimensional metal–insulator transitions. Phys. Rev. Lett. 79, 455 (1997).

[b19] Osofsky M. S. *et al.* Metal-insulator transition in a low mobility two-dimensional system. Accepted for publication in *Scientific Reports*.

[b20] Chen J.-H., Cullen W. G., Jang C., Fuhrer M. S. & Williams E. D.. Defect Scattering in Graphene. Phys. Rev. Lett. 102, 236805 (2009). 19658959 10.1103/PhysRevLett.102.236805

[b21] Bostwick A. *et al.* Quasiparticle Transformation during a Metal-Insulator Transition in Graphene. Phys. Rev. Lett. 103, 056404 (2009). 19792520 10.1103/PhysRevLett.103.056404

[b22] Withers F., Dubois M. & Savchenko A. K.. Electron properties of fluorinated single-layer graphene transistors, Phys. Rev. B 82, 073403 (2010).

[b23] Skrypnyk Y. V. & Loktev V. M.. Metal-insulator transition in hydrogenated graphene as manifestation of quasiparticle spectrum rearrangement of anomalous type, Phys. Rev. B 83, 085421 (2011).

[b24] Lara-Avila S. *et al.* Disordered Fermi Liquid in Epitaxial Graphene from Quantum Transport Measurements. Phys. Rev. Lett. 107, 166602 (2011). 22107411 10.1103/PhysRevLett.107.166602

[b25] Jouault B. *et al.* Interplay between interferences and electron-electron interactions in epitaxial graphene. Phys. Rev. B 83, 195417 (2011).

[b26] Baker A. M. R. *et al.* Weak localization scattering length in epitaxial, and CVD graphene. Phys. Rev. B 86, 235441 (2012).

[b27] Pezzini S., Cobaleda C., Diez E. & Bellani V.. Disorder and de-coherence in graphene probed by low temperature magneto-transport: weak localization and weak antilocalization. J., Journal of Physics: Conference Series 456, 012032 (2013).

[b28] Mahmood A. *et al.* Epitaxial graphene morphologies probed by weak (anti)-localization. J. Appl. Phys. 113, 083715 (2013).

[b29] Chen Y.-F. *et al.* Magnetoresistance in single-layer graphene: weak localization and universal conductance fluctuation studies. J. Phys.: Condens. Matter 22, 205301 (2011). 21393703 10.1088/0953-8984/22/20/205301

[b30] Chen Y.-F. *et al.* Negative and positive magnetoresistance in bilayer graphene: Effects of weak localization and charge inhomogeneity. Physica B 406, 785–788 (2011).

[b31] Jobst J., Waldmann D., Gornyi I. V., Mirlin A. D., Weber H. B. & H. B. Electron-Electron Interaction in the Magnetoresistance of Graphene. Phys. Rev. Lett. 108, 106601 (2012). 22463434 10.1103/PhysRevLett.108.106601

[b32] Gorbachev R. V., Tikhonenko F. V., Mayorov A. S., Horsell D. W., Savchenko A. K. & A. K. Weak Localization in Bilayer Graphene. Phys. Rev. Lett 98, 176805 (2007). 17501523 10.1103/PhysRevLett.98.176805

[b33] Lee P. A. & Ramakrishnan T. V.. Disordered electronic systems. Rev. Mod Phys. 57, 287–337 (1985).

[b34] Belitz D. & Kirkpatrick T. R.. The Anderson–Mott transition. Rev. Mod. Phys. 66, 261–380 (1994).

[b35] McMillan W. L.. Scaling theory of the metal–insulator transition in amorphous materials. Phys. Rev. B 24, 2739–2743 (1981).

[b36] It should be noted that this model is not rigorously correct due to the use of the density of states in the conductance in scaling relations for the renormalization group. However, it has proven to be a useful model for analyzing experimental data (see reference 33).

[b37] Nyakitit L. O. *et al.* Enabling Graphene-Based Technologies: Toward Wafer-Scale Production Of Epitaxial Graphene. MRS Bulletin 37, 1149–1157 (2012).

[b38] Ponomarenko L. A. *et al.* Tunable metal–insulator transition in double-layer graphene heterostructures, Nature Physics 7, 958 (2011).

[b39] Kalon G., Shin Y. J. & Yang H.. Tunable metal–insulator transitions in bilayer graphene by thermal annealing, Appl. Phys. Lett. 98, 233108 (2011).

[b40] Nicotra G., Ramasse Q. M., Deretzis I., La Magna A., Spinella C. & Giannazzo F.. Delaminated Graphene at Silicon Carbide Facets: Atomic Scale Imaging and Spectroscopy, ACS Nano 7, 3045 (2013). 23530467 10.1021/nn305922u

[b41] Hernandez S. C. *et al.* Chemical gradients on graphene to drive droplet motion, ACS Nano 7, 4746–4755 (2013). 23659463 10.1021/nn304267b

[b42] Hernández S. C., Bezares F. J., Robinson J. T., Caldwell J. D., Walton S. G. & S. G. Controlling the local chemical reactivity of graphene through spatial functionalization. Carbon 60, 84–93 (2013).

[b43] Kravchenko S. V. & Sarachik M. P.. Metal–insulator transition in two-dimensional electron systems. Rep. Progr. Phys. 67, 1–44 (2004).

[b44] Fix T., Schoofs F., MacManus-Driscoll J. L. & Blamire M. G.. Charge Confinement and Doping at LaAlO_3_/SrTiO_3_ Interfaces. Phys. Rev. Lett. 103, 166802 (2009). 19905715 10.1103/PhysRevLett.103.166802

[b45] Breckenfeld E. *et al.* Effect of Growth Induced (Non)Stoichiometry on Interfacial Conductance in LaAlO_3_/SrTiO_3_, Phys. Rev. Lett. 110, 196804 (2013). 23705735 10.1103/PhysRevLett.110.196804

[b46] Wong F. J., Chopdekar R. V. & Suzuki Y.. Disorder and localization at the LaAlO_3_/SrTiO_3_ heterointerface. Phys. Rev. B, 82, 165413 (2010).

[b47] Altshuler B. L. & Aronov A. G.. In Electron-Electron Interactions in Disordered Systems (eds Efros A. L. & Pollak M.) 1–154 (North Holland, 1985).

[b48] Bishop D. J., Tsui D. C., Dynes R. C. & R. C. Nonmetallic conduction in electron inversion layers at low temperatures. Phys. Rev. Lett. 44, 1153–1156 (1980).

[b49] Minkov G. M. *et al.* The conductivity of disordered 2D systems: from weak to strong localization. 10^th^ International Symposium on Nanostructures: Physics and Technology, Zhores I. Alferov, L. E., Editors, *Proceedings of SPIE* **5023**, 482–485 (2003).

[b50] Nishida N. *et al.* Transport properties of amorphous Si_1–x_Au_x_: Metal–insulator transition and superconductivity. J. Non-Cryst. Solids 59 & 60, 149–152 (1983).

[b51] Dolan G. J. & Osheroff D. D..“Nonmetallic conduction in thin metal films at low temperatures. Phys. Rev. Lett. 43, 721–724 (1979).

[b52] Abraham D. & Rosenbaum R.. Localization in thin copper films. Phys. Rev. B 27, 1409–1416 (1983).

[b53] Beutler D. E. & Giordano N.. Localization and electron–electron interaction effects in thin Bi wires and films. Phys. Rev. B 38, 8–19 (1988). 10.1103/physrevb.38.8 9945157

[b54] Van den dries L., Van Haesendonck C., Bruynseraede Y. & Deutscher G.. Two-Dimensional Localization in Thin Copper Films, Phys. Rev. Lett., 46, 565–568 (1981).

[b55] Osofsky M., Tardy H., LaMadrid M. & Mochel J. M.. Strong and weak electron spin-orbit scattering near the metal–insulator transition, Phys. Rev. B 31, 4715–4717 (1985). 10.1103/physrevb.31.4715 9936426

[b56] Rosenbaum T. F., Andres K., Thomas Q. A. & Lee P. A.. Conductivity cusp in a disordered metal. Phys. Rev. Lett. 46, 568–571 (1981).

[b57] Paalanen M. A., Rosenbaum T. F., Thomas G. A. & Bhatt R. N.. Stress tuning of the metal–insulator transition at millikelvin temperatures. Phys. Rev. Lett. 48, 1284–1287 (1982).

[b58] Thomas G. A., Paalanen M. & Rosenbaum T. F. Measurements of conductivity near the metal–insulator critical point. Phys. Rev. B 27, 3897–3900 (1983).

[b59] Altshuler B. L. & Aronov A. G.. Fermi-Liquid Theory of the Elelctron-Electron Interaction Effects in Disordered Metals. Sol. St. Commun. 46, 429–435 (1983).

[b60] Castellani C., Di Castro C., Lee P. A. & Ma M.. Interaction-driven metal-insulator transitions in disordered fermion systems. Phys. Rev. B 30, 527–543 (1984).

[b61] Finkelshtein A. M.. Spin fluctuations in disordered systems near the metal–insulator transition. *Zh. Eksp. Teor. Fiz. Pis’ma Red.* **40**, 63 (1984) [*Sov. Phys. JETP Lett.* **40**, 796 (1984)].

[b62] Kirkpatrick T. R. & Belitz D.. Existence of a phase transition in Finkelshtein’s model for a disordered Fermi liquid. Phys. Rev. B 40, 5227–5230 (1989). 10.1103/physrevb.40.5227 9992537

[b63] Fukuyama H. J.. Hall-effect in two-dimensional disordered-systems, Phys. Soc. Jpn. 49, 644–648 (1980).

[b64] Uren M. J., Davies R. A. & Pepper M.. The observation of interaction and localization effects in a two-dimensional electron gas at low temperatures. J. Phys. C: Solid St. Phys. 13, L985–93 (1980).

[b65] McCann E., Kechedzhi K., Fal’ko V., Suzuura H., Ando T. & Altshuler B. L.. Weak-Localization Magnetoresistance and Valley Symmetry in Graphene. Phys. Rev. Lett. 97, 146805 (2006). 17155283 10.1103/PhysRevLett.97.146805

[b66] Gornyi I. V. & Mirlin A. D.. Interaction-induced magnetoresisitance in a two-dimensional electron gas. Phys. Rev. B 69, 045313 (2004).

