# Supplementary material for: Functionalized graphene as a model system for the two-dimensional metal-insulator transition
Source: Sci Rep. 2016 Feb 10;6:19939. doi: 10.1038/srep19939 (PMC4748216; doi:10.1038/srep19939)
Supplement: Supplementary Information [file srep19939-s1.pdf]

Supplementary Information

# Functionalized graphene as a model system for the two-dimensional metal-insulator transition

M. S. Osofsky, S. C. Hernández, A. Nath, V. Wheeler, S. G. Walton, C. M. Krowne, and D. K. Gaskill

Naval Research Laboratory

*1.* *Graphene Growth.* Epitaxial graphene (EG) samples were synthesized by Si sublimation on the nominally on-axis, (0001) (also called the Si-face) of 8 x 8 $mm^2$ semi insulating (>$10^9$ Ωcm) 6H-SiC substrates (II-VI, Inc.) in a commercial Aixtron VP508 chemical vapor deposition reactor. Prior to growth, samples underwent an *in situ* $H_2$ etch, using palladium purified gas, at 1520°C at 100mbar for 10 to 25 minutes to remove any surface damage thus producing a uniform bilayer stepped surface. Graphene formation followed at a temperature of about 1540°C, in a high purity argon atmosphere at 100 mbar for 25 to 35 minutes (*1*).

*2.* *Device Fabrication.* Hall bars were lithographically patterned on the EG by traditional photolithography using LOR and S1811 photoresists to create a clean graphene surface, as described elsewhere(*2*) and this subsequently ensures relatively low metal contact resistance. This process produced atomically smooth samples with clean surfaces (Figs. S1(b)-(d)). The resulting Hall bars were 10 μm wide and 110 μm long, with Ti/Au contacts (10 nm/100 nm). External leads of Au wire connecting the device to a sample holder were added using a K&S Ball Bonder (Model 4522) (Fig S1(a)).

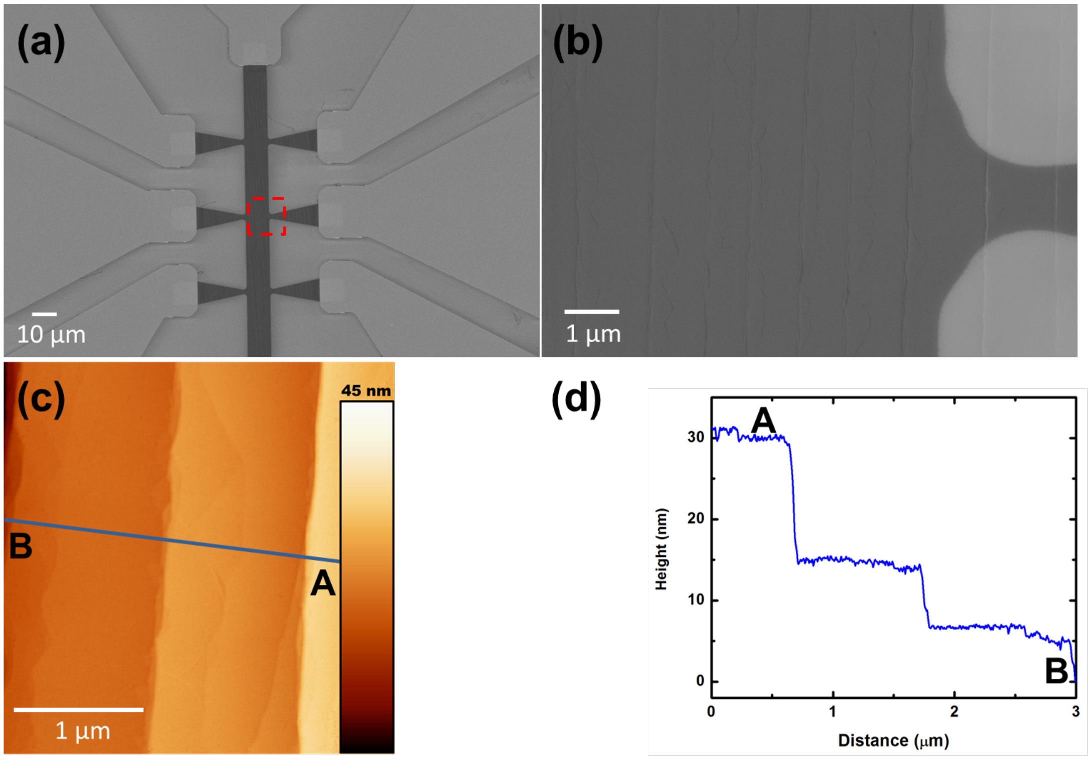


Figure: S1(a) In-lens Scanning Electron Microscope image of a representative Hall structure. (b) Magnified In-Lens SEM image of the boxed region of (a) to demonstrate clean surface after device fabrication. (c) AFM height image of the same device showing the terrace and step structure of the samples. The rms roughness of a 0.5 × 0.5 μm$^2$ region on a terrace is about 0.18±0.03 nm, which is similar to the as-grown sample roughness measured in the same fashion. (d) Line scan from (c) showing typical step heights.

*3.* *Plasma Processing.* While various plasma sources have been used in the synthesis and modification of graphene (*3*), electron beam generated plasmas are well-suited for chemical functionalization, as they are capable of delivering a flux of reactive species while limiting the ion kinetic energies to a few eV (*4*) - a value that is at or near the carbon-carbon bond strength. Thus, they provide the ability to chemically modify graphene without etching or introducing unwanted changes (*5,6*) For this work, pulsed, electron beam driven plasmas were produced in mixtures of $N_2$/Ar, $O_2$/Ar, or $SF_6$/Ar to generate the desired functionalities. High-energy electron beams were created by applying a - 2 kV pulse to a linear hollow cathode for a duration of 2ms at a duty factor of 10%. The emergent beam passed through a slot in a grounded anode and was then terminated at a second grounded anode located further downstream. The electron beam was magnetically confined to minimize spreading via collisions with the background gas, producing a sheet-like plasma. The system base pressure was maintained at ~1x10$^{-6}$ Torr prior to processing by a turbo molecular pump. Reactive gases were introduced at 5% of the total flow rate with argon providing the remainder. The operating pressure (25-90mTorr) was

controlled by adjusting the total flow rate (100-180 sccm). The EG samples were placed on a processing stage adjacent to the plasma at a distance of 2.5 cm from the electron-beam axis. All processing experiments were performed at room temperature. For consistency, a single sample was used for each gas mixture and subjected to repeated plasma treatments and measurements. As such, the reported material properties of any one particular exposure (dose) is the culmination of that exposure plus any prior exposures.

*4.* *Plasma Dose.* It is difficult to precisely know the fluence of reactive species delivered to the graphene surface across a range of operating conditions and background gases. However, since high-energy beam electrons are the primary driver of species production, it is possible to use the processing parameters along with a few assumptions, to estimate the dose of plasma-produced species at the surface (*7*) and compare the results for the various processing conditions. In particular, a comparison of the total production of the primary ionization product in each background gas for a given set of operating parameters serves as a reasonable proxy for the dose of reactive species. Table S1 shows the relative cumulative dose for samples processed under the conditions listed. The notation F1-3, O1-2, and N1-7 refer to sequential exposure to plasmas produced in backgrounds containing $SF_6$, $O_2$, and $N_2$, respectively. Samples labeled O3, O4 and N8 were subject to a vacuum anneal rather than plasma exposure and since annealing effectively removes functional groups, dose is meaningless and thus is omitted. Samples labeled as N0, O0 and F0 refer to the pristine- unfunctionalized devices.

Table S1. Process conditions and relative dose for the samples studied in this work. F, O, and N refer to operating backgrounds containing $SF_6$, $O_2$, and $N_2$, respectively.

| Reactive Background Gas | Sample | Operating Pressure (mTorr) | Plasma Exposure Time (sec) | Cumulative Dose (a.u.) | Vacuum Anneal |
|---|---|---|---|---|---|
| $SF_6$ | F1 | 50 | 6 | 6.63 | - |
| | F2 | 50 | 6 | 9.34 | - |
| | F3 | 90 | 6 | 13.68 | - |
| $O_2$ | O1 | 50 | 6 | 1.88 | - |
| | O2 | 75 | 6 | 5.90 | - |
| | O3 | - | - | - | 450 °C, 1 hr |
| | O4 | - | - | - | 600 °C, 3 hr |
| $N_2$ | N1 | 90 | 6 | 2.59 | - |
| | N2 | 90 | 6 | 7.10 | - |
| | N3 | 75 | 6 | 9.80 | - |
| | N4 | 75 | 6 | 12.91 | - |
| | N5 | 90 | 6 | 17.41 | - |
| | N6 | 90 | 12 | 27.42 | - |
| | N7 | 90 | 24 | 47.44 | - |
| | N8 | - | - | - | 300 °C, 2 hr |

5. *Surface Characterization.* Ex-situ surface diagnostics were performed before and immediately after each sequential plasma exposure to determine the starting material quality and chemistry and the changes resulting from plasma treatment. Ex-situ x-ray photoelectron spectroscopy was performed using a Thermo Scientific K-Alpha spectrometer with a monochromatic Al-K (1486.6 eV) source. The measurement spot size was 100 μm at 100 scans and 100 ms dwell time. The XPS measurements were performed on the lithographically patterned 200 by 200 μm Hall structure.

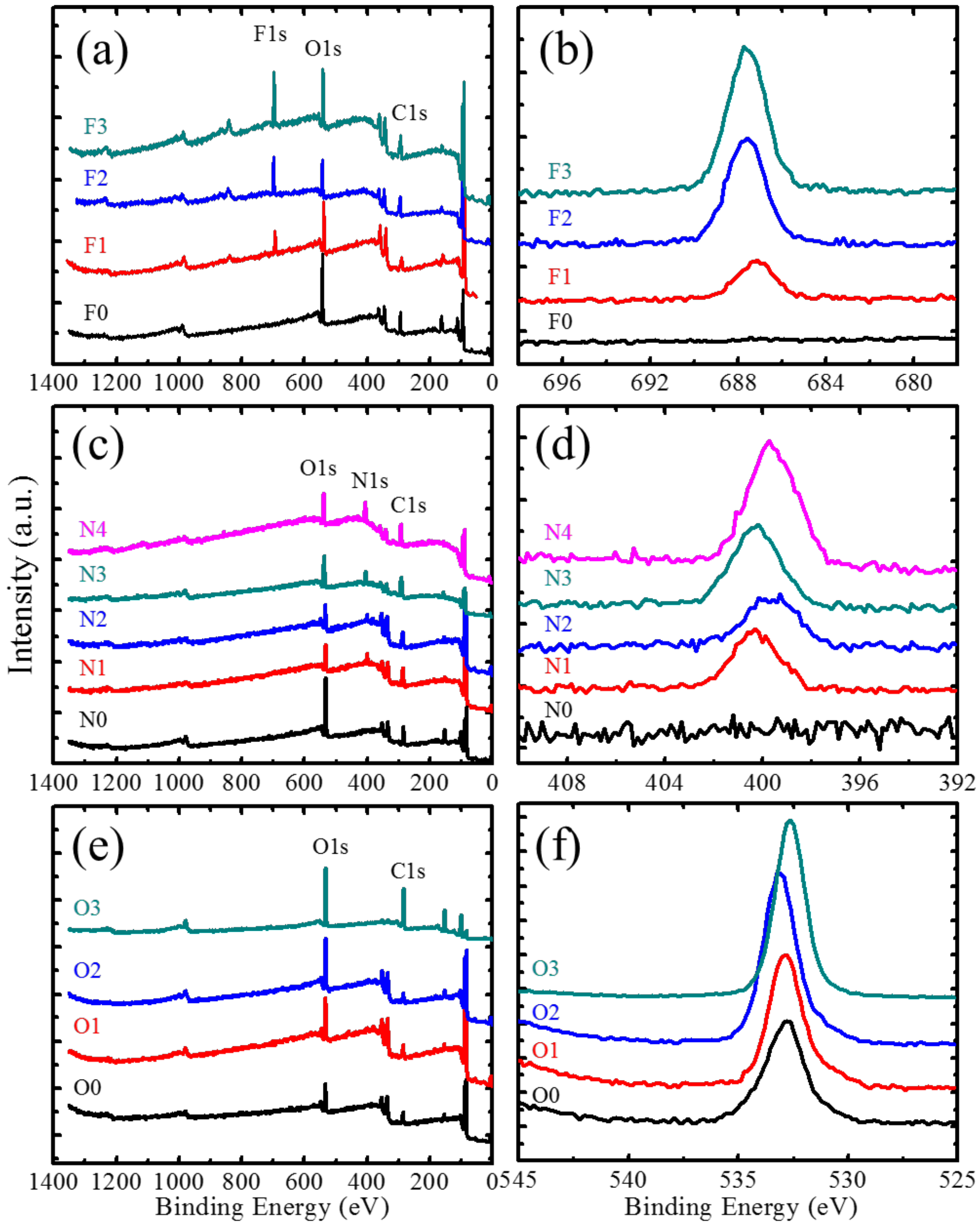


Figure S2: XPS spectra fluorine, nitrogen, and oxygen modified graphene devices. The (a) survey spectra and (b) high resolution F1s spectra for the sequential functionalization of fluorinated devices. (c) Shows the survey and (d) high resolution N1s spectra of the sequential nitrogen functionalization. (e) Shows the survey and (f) and high resolution O1s spectra of oxygen functionalized device.

Chemical changes and the resulting bonding characteristics in the graphene due to plasma processing of individual devices are shown in Figure S2. Following plasma

exposures the XPS survey spectra showed a clear introduction of either fluorine, nitrogen or oxygen species as denoted in Figure S2 (a), (c) and (e), respectively. This introduction of chemical species increased with increasing plasma dose. The high-resolution core level spectra shows the general increase in each curve's intensity demonstrating higher amount of chemical species for each type (F, N or O) (Fig. S2 (b), (d) and (f)). Slight shifts in the spectra could be associated with sample charging due to the insulating nature of the SiC and to the limited graphene material (200 μm x 200 μm). Oxygen peaks present on the survey spectra prior to functionalization could be due to small inadvertent sampling outside of the graphene mesa, and likely in the form of $SiO_2$. The high resolution Si 2p spectra shows two peaks; possibly due to Si or SiC and $SiO_2$ at 99-100 eV and 103-104 eV, respectively. Figure S2 (a) and (b) show increasing fluorine content with each sequential dose. Based on the peak positions of the F1s, the fluorine functionalities added were C-CF, C-F and C-$F_2$, with the latter increasing in content at higher fluorine dosages. This is evident from the peak position shifts towards higher binding energies. For the oxygen case; after oxygen plasma functionalization, features on the O 1s spectra arose at three different locations corresponding to (Si-O) bonding at ≈534.3eV, ethers or alcohols (C-O-C, C-O, or C-OH) at ≈533.3eV and carbonyl groups (C=O) at ≈532.2eV. For the nitrogen scenario, the assignment of the components of the nitrogen functionalized epitaxial graphene device at various operating conditions was challenging due to the overlapping binding energies of nitrogen and oxygen species with those of the interfacial layer. However based on the combined features of the C1s and N1s high resolution spectra, the identifiable peaks are Si-C, C-C $sp^2$, and interfacial layer, respectively. The N1s corroborates the presence of nitrogen functionalities present primarily in the amide and pyrrolic configurations. Oxygen peaks present on all the survey spectra prior to functionalization could be due to small inadvertent sampling outside of the graphene mesa, and likely in the form of $SiO_2$. The high resolution Si 2p spectra in figure S3 shows two peaks that can be attributed to Si or SiC and $SiO_2$ at 99-100 eV and 103-104 eV, respectively. The peaks remain even after the cumulative functionalization of the device (fluorine shown here), and presumed to be characteristic of the substrate. Importantly, there is an evident increase in chemical species bound to the surface of the individual device with each additional plasma exposure without removal of the graphene back bone. These chemical species are covalently attached to the graphene carbon back bone generating surface defects.

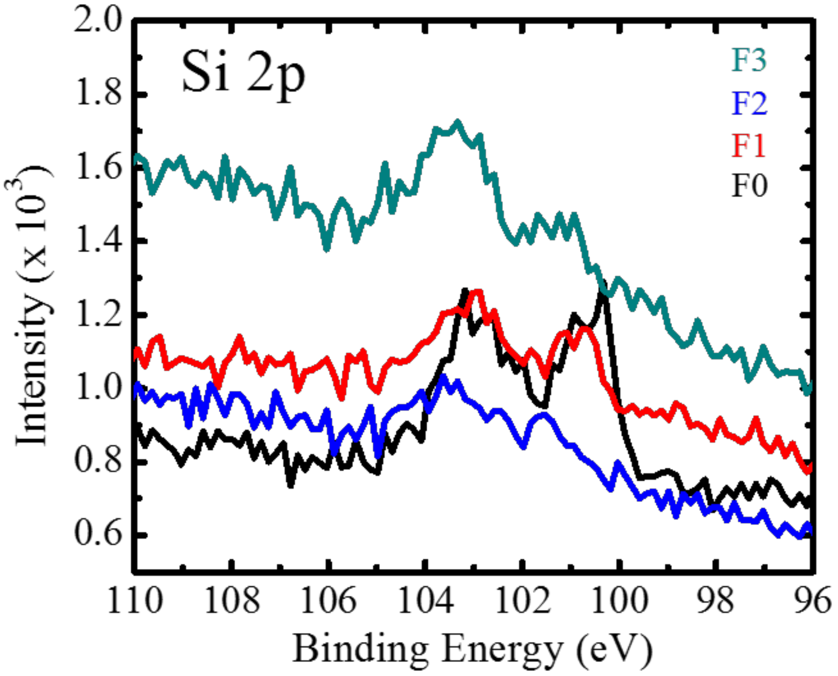


Figure S3: High resolution Si 2p spectra of the fluorinated device before, and after each cumulative fluorine dosage, labeled F0-F3.

Upon annealing, the XPS spectra showed the recovery of the carbon peak to that of $sp^2$ graphene. The XPS C1s spectra showing the chemical recovery of the functionalized graphene device after vacuum annealing (conditions described for O2 and O3 in table 1) is shown in figure S4. The data show that after the second round of oxygen functionalization, the graphene device contained incorporated oxygen functionalities assigned to carbon bonding involving ethers or alcohols (C-O-C, C-O, or C-OH) and carbonyl bonds (=O) located at ≈286.4 eV and ≈287.1eV, respectively. After annealing (O3), the EG C-C $sp^2$ peak intensity increased tremendously, indicating restoration of the graphene lattice upon removal of the oxygen functional groups, clearly showing the partial removal of the attached functional groups and maintained integrity of the graphene $sp^2$ nature.

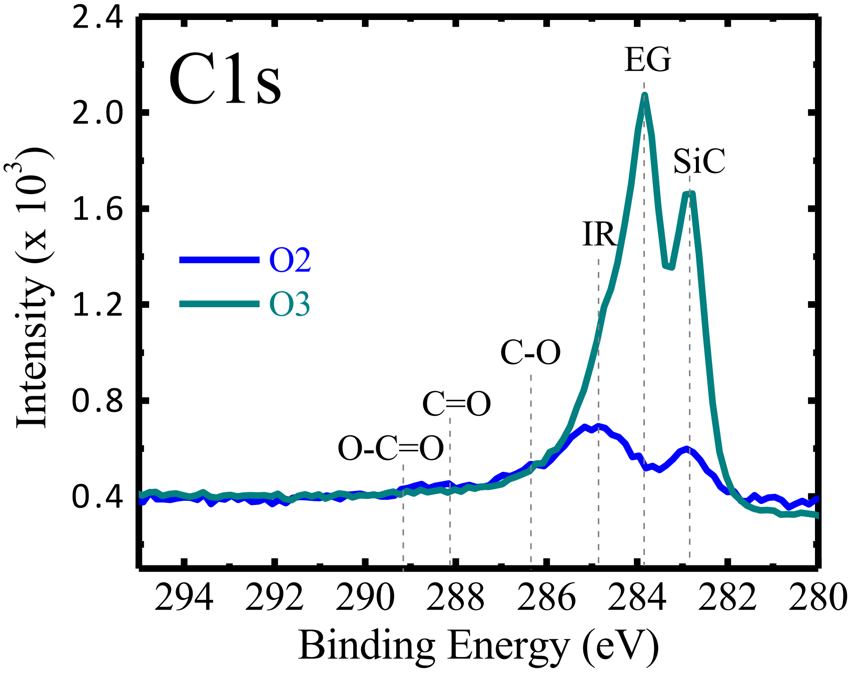


Figure S4: High resolution C1s spectra of an oxygenated device before, O2, and after, O3, vacuum annealing (450°C, 1hr). The graphene peak with $sp^2$ bonding is labeled as EG, the interfacial layer is labeled as IR, and the silicon carbide signal is referred to as SiC.

Raman characterization was performed using an InVia Raman microscope (Renishaw) equipped with a 50x objective lens. A 514.5nm diode laser provided the excitation with the scattered light dispersed by a 1800-line grating into a cooled detector array. Experiments were performed at a set power of 20mW at the source with a spot size of 5μm. Raman spectroscopy is powerful for identifying the number of layers, level of disorder, and doping of graphene. The Raman spectra of pristine and nitrogen functionalized EG devices is shown in Figure S5. Before functionalization there was a weak G peak (≈1600 $cm^{-1}$) and a 2D peak (≈2730$cm^{-1}$), with no detectable D peaks (indicative of EG disorder) for all EG samples. Conjugation of the six member ring structure of graphene can become disrupted when functional groups are introduced to the carbon structure due to electron sharing or $sp^3$-bond formation. Notably, the conversion of the $sp^2$ carbon hybridization to $sp^3$ hybridization breaks symmetry, causing the activation of a breathing mode of the six-membered $sp^2$-carbon rings, which gives rise to a "disorder-induced" peak observed at ~1340 $cm^{-1}$ (D peak) in the Raman spectrum. Therefore, the presence of a D peak in the exposed region is indicative of the localization of defects induced by the incorporation of functional groups in those areas. After plasma treatment, the Raman spectra of the same device showed an increase in the disorder induced D peak and decreases in both the G and 2D peaks,

which is characteristic of the disruption of $sp^2$ bonding of the graphene lattice. The intensity of the D line increased further at higher dosages due to the increased defect sites and decreased $sp^2$ cluster size. However, the 2D peak was still evident even at the highest dosage of functionalization, demonstrating the presence of graphene.

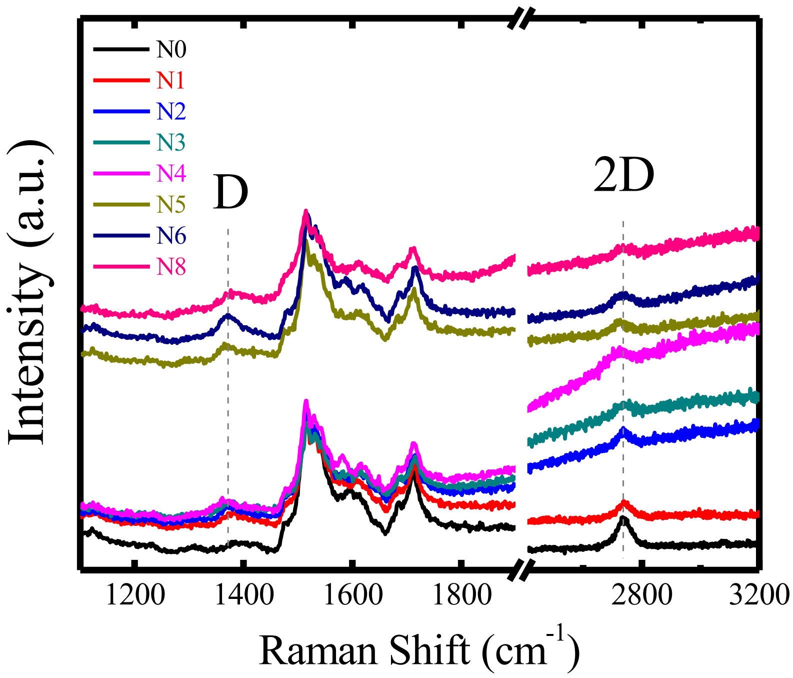


Figure: S5. Raman spectra of epitaxial graphene devices before and after nitrogen functionalization (N0 – N6), and after annealing.

Surface morphology was characterized by Atomic force microscopy (AFM, Bruker Dimension Icon) and scanning electron microscopy (SEM, Carl Zeis).

6. *Influence of plasma functionalization on transport properties*. Standard four-probe resistance and Hall effect measurements were performed on a physical property measurement system (ppms) by Quantum Design. Functionalization through plasma exposure produces scattering sites on the graphene that profoundly affects the resistance. The room temperature resistance rapidly increased with dose and saturated at approximately 8000Ω/square independent of the chemical nature of the dose (Fig. S6(a)). Hall measurements indicate that this increase is predominately due to decreased mobility although there is large scatter in the data for carrier concentration (Fig. S6(b) and (c)). The scatter may be due to variations in the position of the Dirac point which we are unable to determine in this study.

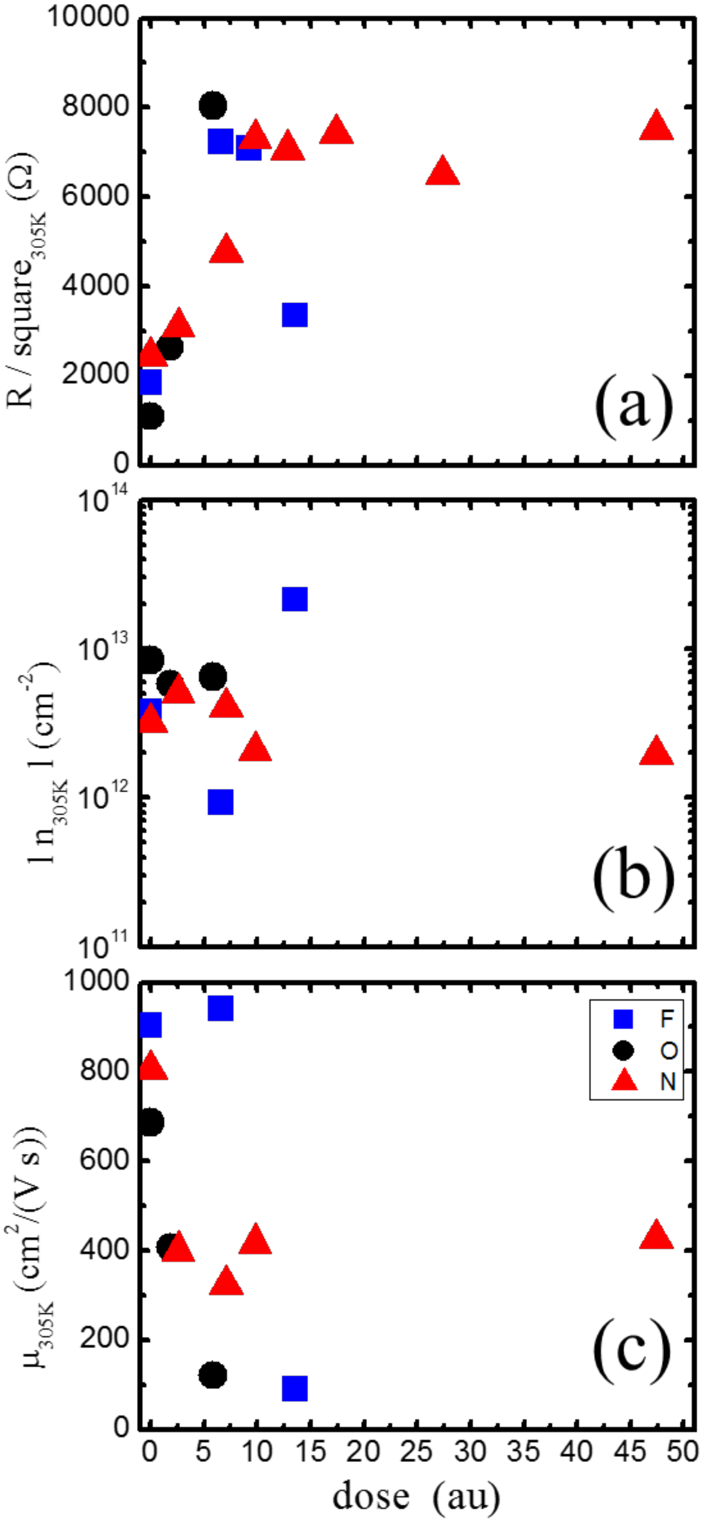


Figure S6. Room temperature transport properties as a function of plasma exposure: (a) resistance, (b) carrier density, and (c) mobility. F, O, and N refer to operating backgrounds containing $SF_6$, $O_2$, and $N_2$,

respectively. The horizontal axis is the same in all cases and can be seen in (c). The results exclude samples subject to vacuum anneals since heating will remove functional groups introduced during plasma exposure.

7. *Heating effects*. It is well known that in conducting systems at low temperatures the energy transfer from electrons to the lattice can be limited by an electron-phonon bottleneck (*8*, *9*, *10*, *11*, *12*, *13*). This phenomenon causes the decoupling of the electrons from the surrounding lattice when a measurement current is applied resulting in a non-equilibrium situation where the electrons have an effective temperature that exceeds that of the surrounding phonon bath. Experimentally, this steady-state manifests as a constant resistivity at low temperature because the electron temperature no longer tracks the measured ambient phonon temperature. This behavior is seen in Fig. S7 where the resistivity of a single sample obtained for the low measurement currents follows a logT dependence while that for the higher measurement currents saturate at values that decrease with increasing measurement current at low temperatures, consistent with the expectation that higher input power results in hotter carriers. The importance of this effect in graphene was noted by Baker, *et al.* (*14* ) This result appears similar to that expected for the Kondo effect which has been reported in graphene with defects (*15*). Also, Price, *et al.* (*16*) reported heating effects below 70K in exfoliated graphene flakes that were attributed to an electron-phonon bottleneck at the graphene substrate interface.

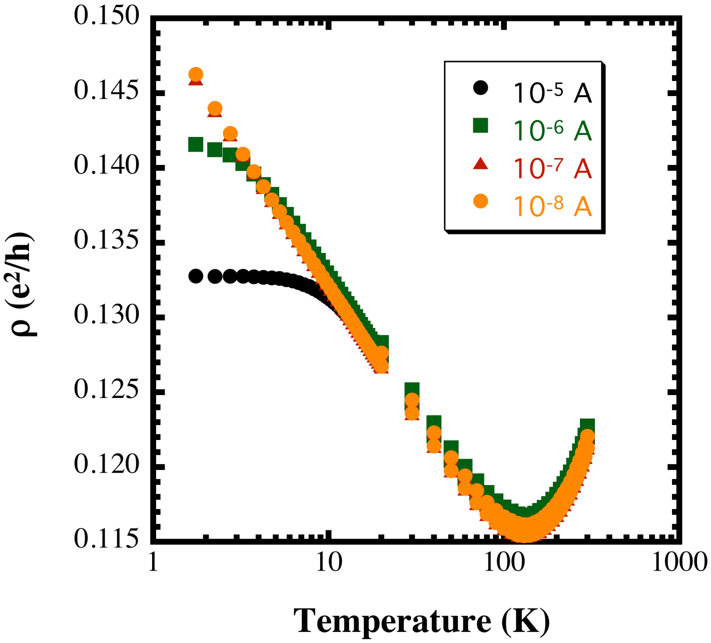


Figure S7. Sheet resistance vs. Temperature using several measurement currents for a sample exposed to $N_2$-containing plasma (sample N1). Note that saturation of sheet resistance occurs with higher measurement currents.

8. *Temperature dependence of the Hall resistance*. Hall resistance data were obtained using a standard Hall bar geometry for -8T≤B≤8T in a Quantum Design Physical Property Measurement System (PPMS). The data for negative B were subtracted from those for positive B and averaged to remove the contribution from the magneto-resistance. The low temperature $R_{Hall}$ values for several plasma doses (see Table S1 for dose details; 0 indicates unexposed samples) are normalized to the $R_{Hall}$ values at 1.75K and are plotted as a function of log(T) in Fig. S8. These results demonstrated that like $R_{square}$, $R_{Hall} \sim \log T$.

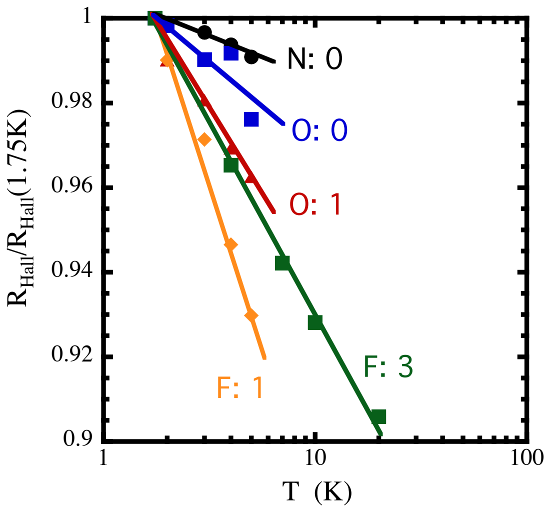


Figure S8. Normalized Hall coefficient vs. log(T) for several samples and plasma doses at low temperature. The lines are guides for the eye. The exposures are shown in table S1.

9. *Relaxation times*. The decoherence (a), intervalley (b), and multicomponent (c) relaxation times extracted from fits of equation 3 in the main text to the magneto-resistance (MR) data are shown as a function of σ(1K), a measure of the distance from the strongly localized phase are shown in Fig. S9. It is important to note that the theory was developed for systems far from the strongly localized state while these samples were very close and that one should be wary of taking the values of these times too seriously (see main text). However, these results can give us some insight into the nature of the type disorder that is responsible for the localization behavior. It is believed that electronic transport in graphene is sensitive to the type of disorder which can be due to short-range or long-range defects, neutral or charged impurities, adsorbed or interstitial atoms or molecules, vacancies, spatial distortions, or structural irregularities, including ripples on the graphene sheet and other long-wave random modulations (17). However, the original models of localization are very general and only require a disordered potential (e.g. E. Abrahams, *et al.* (18)). Since those models successfully account for the observed σ(T), those results cannot distinguish between the types of disorder. However, the

relaxation times extracted from the analysis of the magneto-resistance does give information on the relevant scattering channels and thus the type of disorder. From figure S9 it is apparent that $\tau_*$ has the smallest values. We can extract information concerning the dominant disorder contribution since $\tau_*$ is defined as $\tau_*^{-1} = \tau_w^{-1} + \tau_z^{-1} + \tau_i^{-1}$, where $\tau_w$ is the relaxation time associated with the warping term, $\tau_i$ is the intervalley relaxation time, and $\tau_z$ is the intravalley relaxation time. Since figure S9(b) shows that $\tau_i^{-1}$ is negligible, we find that the warping and/or intravalley disorder dominate.

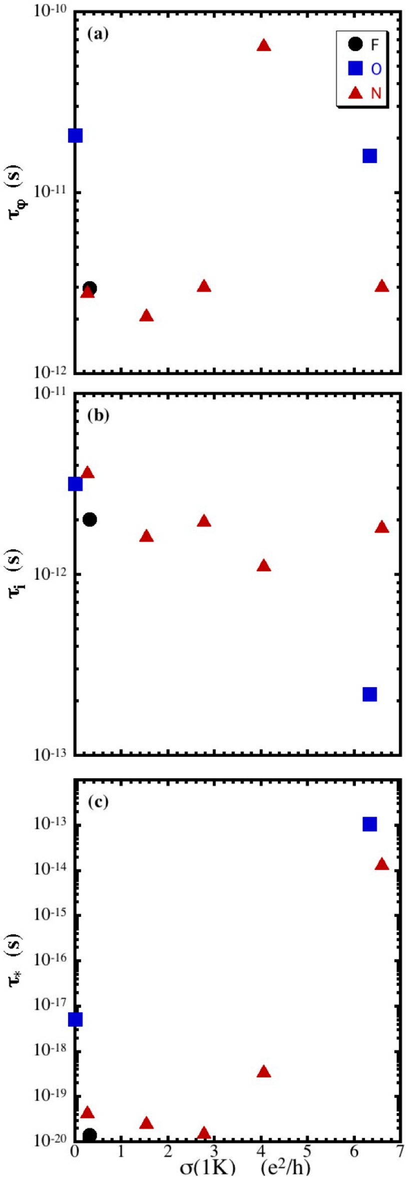


Figure S9. Characteristic time scales extracted from the MR data: (a) decoherence, (b) intervalley, and (c) $\tau_*$, defined as $\tau_*^{-1} = \tau_w^{-1} + \tau_z^{-1} + \tau_i^{-1}$, where $\tau_w$ is the relaxation time associated with the warping term and $\tau_z$ is the intravalley relaxation time. The horizontal axis for (a), (b) and (c) are the same and is found in (c).

1. L. O. Nyakitit, *et al.*, Enabling Graphene-Based Technologies: Toward Wafer-Scale Production Of Epitaxial Graphene. MRS Bulletin **37**, 1149-1157 (2012).
2. A. Nath, *et al.*, Achieving clean epitaxial graphene surfaces suitable for device applications by improved lithographic process. *Appl. Phys. Lett.* **104**, 224102 (2014).
3. K. Ostrikov, E. C. Neyts, M. Meyyappan, Plasma nanoscience: from nano-solids in plasmas to nano-plasmas in solids. Advances in Physics 62 (11), pp. 113-224 (2013).
4. S. G. WaltonC. Muratore, D. Leonhardt, R. F. Fernsler,D. D. Blackwell, R. A. Meger, Electron beam-generated plasmas for materials processing. Surface and Coatings Technology 186, 40-46 (2004).
5. M. Baraket, S. G. Walton, E. H. Lock, J. T. Robinson, F. K. Perkins, The functionalization of graphene using electron-beam generated plasmas. Appl. Phys. Lett. **96**, 231501 (2010).
6. S. C. Hernandez, *et al.*, Chemical gradients on graphene to drive droplet motion. ACS Nano **7**, 4746-4755 (2013).
7. S. G. Walton, *et al.*, Study of plasma-polyethylene interactions using electron beam generated plasmas produced in Ar/SF6 mixtures. J. Appl. Polym. Sci. **117**, 3515-3523 (2010).
8. G. J. Dolan, D. D. Osheroff, Nonmetallic conduction in thin metal films at low temperatures. *Phys. Rev. Lett.* **43**, 721–724 (1979).
9. D. J. Bishop, D. C. Tsui, R. C. Dynes, Nonmetallic conduction in electron inversion layers at low temperatures. *Phys. Rev. Lett.* **44**, 1153–1156 (1980).
10. H. Hoffmann, F. Hofmann, F. & W. Schoepe, Magnetoresistance and non-Ohmic conductivity of thin platinum films at low temperatures, *Phys. Rev. B* **43**, 5563 (1982).
11. G. Bergmann, Influence of electric field on weak localization. Zeitschrift fur Physik B **49**, 133 (1982).
12. P. W. Anderson, E. Abrahams, T. V. Ramakrishnan, Possible Explanation of Nonlinear Conductivity in Thin-Film Metal Wires. *Phys. Rev. Lett.* **43**, 718 (1979).
13. J. F. DiTusa, K. Lin, M. Park, M. S. Isaacson, J. M. Parpia, Role of Phonon Dimensionality in Electron-Phonon Scattering Rates. *Phys. Rev. Lett.* **68**, 1156 (1992).
14. A. M. R. Baker, *et al.*, Weak localization scattering lengths in epitaxial, and CVD graphene. Physical Review B 86, 235441 (2012).
15. J.-H. Chen, L. Li, W. G. Cullen, E. D. Williams, M. S. Fuhrer, Tunable Kondo effect in graphene with defects. Nature Physics **7**, 535 (2011).
16. A. S. Price, S. M. Hornett, A. V. Shytov, E. Hendry, D. W. Horsell, Nonlinear resistivity and heat dissipation in monolayer graphene. *Phys. Rev. B* **85**, 161411 (2012).
17. Y.V. Skrypnyk, and V.M. Loktev, Metal-insulator transition in hydrogenated graphene as manifestation of quasiparticle spectrum rearrangement of anomalous type, Phys. Rev. B **83**, 085421 (2011).

18. E. Abrahams, P. W. Anderson, D. C. Licciardello, T. V. Ramakrishnan, Scaling theory of localization: Absence of quantum diffusion in two dimensions. *Phys. Rev. Lett.* **42**, 673–676 (1979).
